# 17β-Estradiol Counteracts Pathological Microtubule Remodeling To Enhance Cardiac Function

**DOI:** 10.1101/2025.01.22.634271

**Published:** 2025-01-24

**Authors:** Ryan Moon, Neal T. Vogel, Jenna B. Mendelson, Lynn M. Hartweck, John P. Carney, Minwoo Kim, Melissa K. Gardner, Sasha Prisco, Kurt W. Prins

**Affiliations:** 1. Lillehei Heart Institute, Cardiovascular Division, University of Minnesota, Minneapolis, MN; 2. Department of Integrative Biology and Physiology, University of Minnesota, Minneapolis, MN; 3. Experimental Surgical Services, Department of Surgery, University of Minnesota, Minneapolis, MN; 4. Department of Genetics, Cell Biology, and Development, University of Minnesota, Minneapolis, MN

## Abstract

The female-predominate sex hormone 17β-estradiol exerts cardioprotective effects via multiple mechanisms. Available data demonstrate 17β-estradiol modulates microtubule dynamics *in vitro*, but its effects on pathogenic microtubule remodeling in pressure-overloaded cardiomyocytes are unexplored. Here, we show 17β-estradiol directly blunts microtubule polymerization *in vitro*, counteracts endothelin-mediated microtubule remodeling in iPSC-cardiomyocytes, and mitigates microtubule stabilization in pulmonary artery banded right ventricular cardiomyocytes. 17β-estradiol treatment blunts cardiomyocyte and nuclear hypertrophy, restores t-tubule architecture, and prevents mislocalization of connexin-43 in RV cardiomyocytes of pulmonary artery banded rats. These cellular phenotypes are paired with significant improvements in RV function. Thus, we propose 17β-estradiol exerts cardioprotective effects via direct modulation of microtubules in addition to its well ascribed signaling functions.

Cardiomyocyte microtubule remodeling occurs in multiple species challenged by pressure overload, and heightened microtubule density promotes cardiomyocyte dysfunction^1^. The microtubule cytoskeleton impacts several areas of cardiac biology by regulating cardiomyocyte hypertrophy, nuclear remodeling, t-tubule structure, and the proper localization of the gap junction protein connexin-43^1^. In addition, cross-talk between the microtubule cytoskeleton and the desmin intermediate filament cytoskeleton regulates some of the microtubule-dependent cardiomyocyte phenotypes^1^. 17β-estradiol, the female-predominant hormone, induces microtubule depolymerization in breast cancer cells lacking estrogen receptors^2^. *In vitro*, 17β-estradiol slows microtubule polymerization rates^3^, which demonstrates 17β-estradiol directly regulates microtubule dynamics. While 17β-estradiol exerts cardioprotective effects via pleotropic mechanisms^4^, its effects on pathological microtubule remodeling in pressure-overloaded cardiomyocytes are unexplored. Here, we investigate the hypothesis that 17β-estradiol suppresses pathological microtubule remodeling, which restores cardiomyocyte/nuclear size, t-tubule morphology, and connexin-43 localization and ultimately enhances cardiac function in pulmonary artery banded rats.

A fluorescent tubulin kit (Cytoskeleton:BK011P) quantified *in vitro* microtubule polymerization rates in the presence of ethanol vehicle or 17β-estradiol. Human induced pluripotent stem cells (iPSC, AICS-0011, Allen Institute) were differentiated into cardiomyocytes (iPSC-CM) as recommended and then iPSC-CM were treated with endothelin (100nM, Tocris 1160) or endothelin+17β-estradiol (100 nM endothelin, 100 nM 17β-estradiol) overnight. iPSC-CMs were fixed with 4% paraformaldehyde, treated with 1% Triton X100, washed/blocked and incubated with primary antibodies to β-tubulin (Abcam:ab6046) or detyrosinated α-tubulin (Abcam:AB48389). Cells were washed, incubated with Alexa-568 secondary antibody, and imaged. Adult male Sprague Dawley rats were subjected to pulmonary artery banding (PAB) using an 18-gauge needle. Two weeks post banding, rats were randomly allocated to daily 17β-estradiol treatment (0.2 mg/kg PAB-E2) or ethanol vehicle (PAB-Vehicle) via intraperitoneal injection for two weeks. Formalin sections of right ventricular (RV) free walls were subjected to heat-mediated antigen retrieval (Reveal Decloaker, Biocare Medical), stained with primary antibodies to β-tubulin (Abcam:AB6046), desmin (ProSci:46-777), connexin-43 (Abcam:AB235585), and then counterstained with AlexaFluor-568 anti-rabbit secondary antibody (ThermoScientific:A-11036), Wheat Germ Agglutinin (WGA) AlexaFluor-488 (ThermoFisher:W21404), and mounted in ProLong Glass with NucBlue Antifade Mountant (ThermoFisherScientific:P36985). Confocal micrographs were collected on a Zeiss LSM900 Airyscan 2.0 microscope. Confocal images were blindly analyzed by RM using FIJI. Echocardiography and invasive closed-chest hemodynamics evaluated rodent right ventricular systolic pressure and RV function^5^. Animal studies were approved by the UMN Institutional Animal Care and Use Committee. Statistical analyses were performed using GraphPad Prism 10.1.

We first demonstrated 17β-estradiol directly impacted microtubule dynamics as 17β-estradiol significantly slowed microtubule polymerization rates *in vitro* (Figure A). Next, we showed the microtubule-suppressing effects of 17β-estradiol were present in human iPSC-CM to provide cardiac-cell relevance to our *in vitro* finding. As compared to control cells, endothelin treatment increased the density of both total and detyrosinated microtubules (Figure B and C). However, 17β-estradiol treatment blunted endothelin-mediated densification of total and detyrosinated microtubules (Figure B and C). Thus, 17β-estradiol reduced microtubule polymerization rates and counteracted chemical stress-induced microtubule remodeling in iPSC-CMs.

Then, we quantified the physiological effects of 17β-estradiol using both echocardiography and right heart catheterization using a translational approach by starting treatment two weeks after pulmonary artery banding to allow for the development of RV dysfunction (Figure D). Echocardiographic analysis revealed 17β-estradiol increased tricuspid plane annular systolic excursion (TAPSE) and right ventricular free wall thickening (Figure E). Importantly, right ventricular systolic pressure was equivalently elevated in PAB-Vehicle and PAB-E2 animals (Figure E). These data suggested 17β-estradiol imparted an inotropic effect independent of RV afterload.

Finally, we determined how 17β-estradiol treatment modulated RV cardiomyocyte microtubule phenotypes in PAB rats to assess if the observed improvements in cardiac function were associated with alterations in the microtubule cytoskeleton. Confocal microscopy revealed a significant increase in microtubule density in RV cardiomyocytes following PAB, which 17β-estradiol mitigated (Figure F). Importantly, 17β-estradiol did not alter desmin localization patterns (Figure G), which implied its effects on cardiac morphology were predominately due to microtubule modulation rather than desmin regulation. The reduction in microtubule density with 17β-estradiol treatment limited both cardiomyocyte and cardiomyocyte nuclear hypertrophy (Figure H). 17β-estradiol also combatted other pathogenic microtubule-associated phenotypes as it improved t-tubule structural integrity, and prevented the lateral membrane localization of connexin-43 (Figure H). Thus, the cardioprotective effects of 17β-estradiol were paired with corrections of multiple microtubule-based phenotypes in RV cardiomyocytes.

In conclusion, we show 17β-estradiol slows microtubule polymerization *in vitro*, and counteracts endothelin-mediated microtubule densification in human iPSC-CM. In PAB rats, 17β-estradiol mitigates microtubule remodeling, and downstream pathogenic phenotypes in RV cardiomyocytes. Importantly, these structural changes occur without significant modulation of desmin localization, which further supports 17β-estradiol’s cellular effects are related to its microtubule-modulating properties. The correction of microtubule-associated cellular phenotypes ultimately manifest as improvements in RV function independent of afterload. In summary, our data further support a pathogenic role of hyperstabilized microtubules in cardiac dysfunction. We therefore nominate the direct modulation of microtubule remodeling as a novel mechanism by which 17β-estradiol exerts cardioprotective effects in addition to its known signaling roles^4^.

## Figures and Tables

**Figure:**
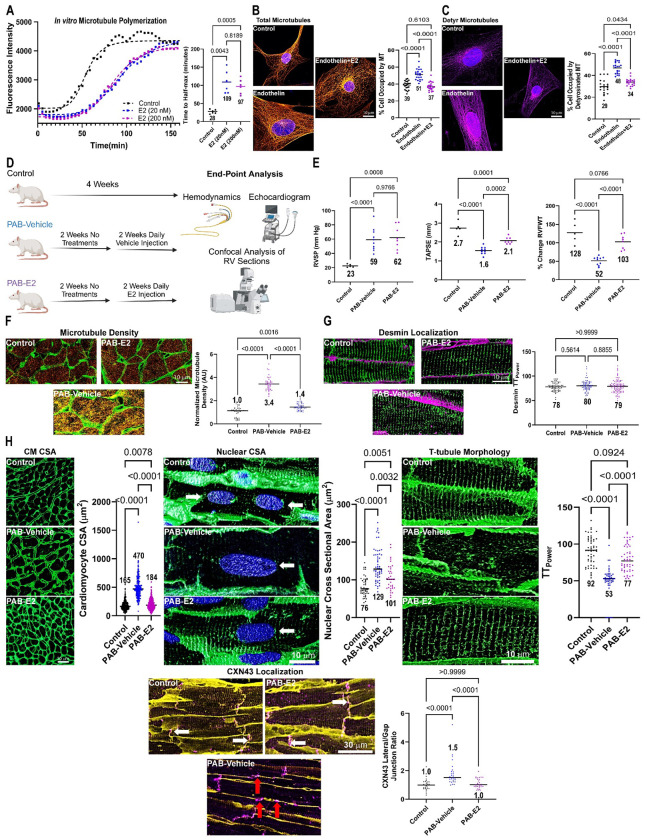
17β-Estradiol Limits Pathological Microtubule Stabilization and Improves Cardiac Function in Pulmonary Artery Banded Rats. (A) Microtubule polymerization kinetics in the presence of absence of 17β-estradiol. Quantification of time to half max in presence of vehicle, 20 nM 17β-estradiol, and 200 nM 17β-estradiol. *p*-values determined by one-way ANOVA with Tukey’s multiple comparisons test. Representative confocal micrographs of iPSC-CM stained with β-tubulin (orange, B), detyrosinated α-tubulin (purple, C), and DAPI (blue). Quantification of total microtubule (B) and detyrosinated microtubule (C) density. *p*-values determined by one-way ANOVA with Tukey’s multiple comparisons test for total microtubule density and Brown-Forsythe ANOVA and Dunnett’s T3 multiple comparisons test for detyrosinated microtubule density. (D) Representation of experimental approach for evaluating *in vivo* microtubule effects of 17β-estradiol. (E) E2 treatment did not reduce right ventricular systolic pressure but increased RV function determined by TAPSE and % change in RV free wall thickness. *p*-values determined by one-way ANOVA with Tukey’s multiple comparisons test for TAPSE and percent RV free wall thickening and Brown-Forsythe ANOVA and Dunnett’s T3 multiple comparisons test for RVSP. (F) WGA (green) and microtubule staining (orange) of RV free wall sections from control, PAB-Vehicle, and PAB-E2 RV sections with quantification of relative microtubule fluorescence intensity. *p*-values determined by one-way ANOVA with Tukey’s multiple comparisons test. (G) Confocal micrographs of RV sections stained with WGA (purple) and desmin (green). Desmin localization patterns were similar in all three experimental groups. *p*-values determined by Kruskal-Wallis and Dunn’s multiple comparisons test. (H) WGA (green) and DAPI (blue) delineated cardiomyocyte and nuclear size (white arrow). *p*-values determined by Kruskal-Wallis test with Dunn’s multiple comparison test. 17β-estradiol restored the normal striated t-tubule architecture in RV sections (*p*-values determined by Kruskal-Wallis test with Dunn’s multiple comparison test). 17β-estradiol prevented lateralization of connexin-43 (purple) in the RV (red arrows: lateralized connexin-43, white arrows: connexin-43 at intercalated disc). *p*-values determined by Kruskal-Wallis test with Dunn’s multiple comparison test.
